# Will I Regret It? Anticipated Negative Emotions Modulate Choices in Moral Dilemmas

**DOI:** 10.3389/fpsyg.2016.01918

**Published:** 2016-12-06

**Authors:** Carolina Pletti, Lorella Lotto, Alessandra Tasso, Michela Sarlo

**Affiliations:** ^1^Department of General Psychology, University of PadovaPadova, Italy; ^2^Department of Developmental Psychology and Socialization, University of PadovaPadova, Italy; ^3^Center for Cognitive Neuroscience, University of PadovaPadova, Italy; ^4^Department of Human Studies, University of FerraraFerrara, Italy

**Keywords:** moral dilemma, emotion, decision-making, readiness potential

## Abstract

We tested if post-decisional emotions of regret, guilt, shame, anger, and disgust can account for individuals’ choices in moral dilemmas depicting the choice of letting some people die (non-utilitarian option) or sacrificing one person to save them (utilitarian option). We collected participants’ choices and post-decisional emotional ratings for each option using Footbridge-type dilemmas, in which the sacrifice of one person is the means to save more people, and Trolley-type dilemmas, in which the sacrifice is only a side effect. Moreover, we computed the EEG Readiness Potential to test if the neural activity related to the last phase of decision-making was related to the emotional conflict. Participants reported generally stronger emotions for the utilitarian as compared to the non-utilitarian options, with the exception of anger and regret, which in Trolley-type dilemmas were stronger for the non-utilitarian option. Moreover, participants tended to choose the option that minimized the intensity of negative emotions, irrespective of dilemma type. No significant relationship between emotions and the amplitude of the Readiness Potential emerged. It is possible that anticipated post-decisional emotions play a role in earlier stages of decision-making.

## Introduction

In the last 15 years, moral dilemmas have been widely employed in psychology and neuroscience to investigate the interplay between emotional and cognitive processes in moral judgment and decision-making. In the *Trolley dilemma*, a runaway railway trolley is about to run over a group of five unaware workers. The only way to save them is to pull a lever and divert the trolley onto another rail, where a single worker stands, who would be run over instead. In the *Footbridge dilemma*, the only way to save the five is to push a large stranger off an overpass, so that his body would stop the trolley. It is a well-known and widely replicated result that individuals usually endorse the choice to sacrifice one person to save five lives in the Trolley dilemma, but not in the Footbridge dilemmas (Greene et al., 2001; Schaich Borg et al., 2006; Hauser et al., 2007; Sarlo et al., 2012).

According to Greene et al. (2001, 2004) dual process model, this pattern of findings is due to the fact that moral judgments and decisions are driven by two systems in competition: a slow, deliberate, and rational system, that would perform a cost-benefit analysis and lead individuals to endorse the option that maximizes the number of spared lives – the so-called *utilitarian* resolution of the dilemmas – and a fast, automatic, and emotional system, that would work like a sort of “alarm bell” producing an immediate negative reaction against the proposed action (i.e., killing a man), leading individuals to reject the utilitarian resolution. According to the dual process model, when the sacrifice of one person is particularly aversive – like in the Footbridge dilemma – the emotional system would prevail over the rational one and push toward the rejection of the utilitarian choice.

Starting from this model, several studies tested the hypothesis that emotional processing plays a major role in moral decisions and judgments, yielding generally consistent results: for instance, the resolution of Footbridge-type dilemmas elicits greater activity in brain areas associated with emotional processing, like the ventromedial prefrontal cortex (vmPFC), the superior temporal sulcus (STS), and the amygdala, as compared to Trolley-type dilemmas (Greene et al., 2004, 2001). Furthermore, individuals with emotional hyporeactivity (e.g., participants with high psychopathy traits, or vmPFC impairments) show a higher endorsement of utilitarian options as compared to control participants (e.g., Koenigs et al., 2007, 2012; Moretto et al., 2010; Pletti et al., 2016).

Given this evidence, we might expect individuals to experience a more negative emotional state while deciding in Footbridge- as compared to Trolley-type dilemmas. Moreover, we might expect that a more intense negative emotional state would be related to a lower percentage of utilitarian choices. However, evidence of an association between self-report ratings of the emotional state experienced by participants and moral judgments or choices has been mixed: of the few studies that collected participants’ emotional evaluations during the task (Choe and Min, 2011; Sarlo et al., 2012; Lotto et al., 2014; Szekely and Miu, 2015; Horne and Powell, 2016), only one (Horne and Powell, 2016) reported an association between emotions and moral judgment, and none reported an association between emotional ratings and choices. In a study by Krosch et al. (2012) that used realistic military scenarios, participants that reported having based their choices on their emotional reaction were more likely to choose a “humanitarian” option (protecting civilians from being attacked) over a “military” one (not acting in order to preserve neutrality). Despite this, however, the differences in the emotional states associated with the two options did not predict the choices. Thus, the relationship between subjectively experienced emotions and decisions in moral dilemmas is still unclear. Shedding light on this aspect would provide important information in understanding how emotion influences moral decisions.

It is important to note that the aforementioned studies (with the exception of Krosch et al., 2012) focused on the immediate emotions that participants experienced at the moment of the decision, and did not examine the role played by anticipating post-decisional emotional consequences. This is a relevant point, because according to several models of decision-making like the regret theory (Bell, 1982; Loomes and Sugden, 1982), the disappointment theory (Bell, 1985; Loomes and Sugden, 1986), and the decision affect theory (Mellers et al., 1999), the anticipation of post-decisional emotions has a crucial impact on decisions. According to these models, during decision-making, individuals try to predict how they would feel after having chosen each of the different alternatives, and then select the option that minimizes the anticipated negative emotions. In particular, individuals are especially motivated to avoid post-decisional feelings of regret, arising when the outcome of the chosen option is worse than what would have resulted from the alternative options, and feelings of guilt, entailing self-condemning feelings elicited by causing harm or distress to others (Tangney et al., 1996; Haidt, 2003). The need to avoid guilt strongly influences decision-making when the individual’s decisions have relevant consequences for others: in particular, guilt aversion motivates individuals to act cooperatively (Ketelaar and Tung Au, 2003; de Hooge et al., 2007; Chang et al., 2011) and to avoid deception (Charness and Dufwenberg, 2006).

It is therefore plausible that in moral dilemmas decisions could be driven by the attempt to minimize post-decisional negative emotions. In line with this hypothesis, it has been reported that the percentage of utilitarian choices endorsed in Footbridge-type moral dilemmas is inversely predicted by individuals’ disposition to experience personal distress when faced with the suffering of others (Sarlo et al., 2014). Furthermore, a recent study investigating post-decisional emotions arising from moral dilemmas suggests that, in Footbridge-type dilemmas only, participants’ choices seem to be aimed at minimizing post-decisional regret (Tasso et al., unpublished). In particular, the greater the regret experienced after the counterfactual decision relative to the regret experienced after the non-utilitarian actual decision, the higher the number of non-utilitarian choices in Footbridge-type dilemmas. However, the above study did not directly compare the emotions related to the utilitarian and non-utilitarian options between the two dilemma types. Thus, it is still not known whether the utilitarian option is indeed associated to higher emotional intensities in Footbridge- than in Trolley-type dilemmas.

The present study aimed at systematically comparing the post-decisional emotions associated with the utilitarian and non-utilitarian options in Footbridge-type and Trolley-type dilemmas, irrespective of participants’ choices, by focusing on whether the difference between the two emotional states could predict participants’ choices. We hypothesized that, if participants anticipate the emotional consequence of a decision and use this information to guide their choices, then the difference between the emotions associated with the two options should predict participants’ choices.

A second, independent, goal of this study was to identify a possible electrophysiological correlate of the conflict between anticipated emotional consequences during the resolution of the dilemmas. We focused on the last phase of the decision-making process, in which an option is selected and the corresponding action is implemented (Ernst and Paulus, 2005), and we analyzed the readiness potential (RP), a slow negative EEG wave that is observed before the execution of a voluntary movement. The RP recorded over the central electrode Cz reflects an increase in the cortical excitability of brain areas involved in the preparation of movement, like the supplementary motor area (SMA) and the pre-motor cortex (Shibasaki and Hallett, 2006). Indeed, besides being involved in the preparation and selection of actions (Rushworth et al., 2004), the SMA plays a crucial role in value-based decision-making, being involved in reward anticipation (Lee, 2004) and in the encoding of the reward value associated with an action (Wunderlich et al., 2009). In line with these functions of the SMA, some recent findings demonstrated that the RP tracks the emergence of value-based decisions and reflects the readiness to provide a response in a decision-making task (Gluth et al., 2013).

The RP also seems to have an important role in moral decisions, since it might reflect the conflict that is inherent in these decisions. Indeed, lower amplitudes of the RP were reported for Footbridge-type as compared to Trolley-type dilemmas, reflecting lower preparation to respond, possibly due to a greater conflict between alternative options (Sarlo et al., 2012). Consistent with this finding, an fMRI study reported greater SMA activation for Trolley-type as compared to Footbridge-type dilemmas (Schaich Borg et al., 2006). Finally, another EEG study found a smaller RP amplitude for deception than for truth telling (Panasiti et al., 2014), which can also be related to moral conflict.

Taken together, these results suggest a relationship between the amplitude of the RP and the intensity of conflict in moral situations. Thus, in the present study we aimed at testing if the conflict reflected in the amplitude of the RP has an emotional nature, and reflects the degree with which individuals comply with their emotional evaluations in making their decisions. To this aim, we measured emotional experiences related to both the chosen and the unchosen option for each dilemma, and we calculated an index of conflict between participants’ decision and their emotional evaluations, testing its association to the RP amplitude.

To summarize, the main aim of the present study was to test the influence of anticipated emotional consequences on decisions in moral dilemmas. We hypothesized that in Footbridge-type dilemmas, but not in Trolley-type dilemmas, the utilitarian option would be associated with more negative emotional consequences as compared to the non-utilitarian option. This would contribute to explain why in Footbridge-type dilemmas people reject the rational option that maximizes the number of saved lives, whereas in Trolley-type dilemmas people endorse it. Furthermore, we hypothesized that participants would choose the option associated with the lowest emotional cost, and we expected this effect to emerge for Footbridge-type dilemmas only, since in Trolley-type dilemmas the emotional cost between options is not so different to critically influence decisions.

We focused on different emotions: guilt and shame, which are two of the most prototypical moral emotions (Haidt, 2003); disgust and anger, which are two basic emotions typically elicited by moral violations (Haidt, 2003); and finally, regret, which is a crucial decision-related emotion elicited by comparing the outcome of choices. For each dilemma, we collected self-report measures of the emotional state experienced by participants relative to both the utilitarian and the non-utilitarian option, and we tested whether participants chose the option associated with the lower emotional cost. Finally, we recorded the RP time-locked to the moment of choice, to test whether it reflects an emotional conflict developing during the decision-making.

## Materials and Methods

### Participants

Fifty-six healthy participants aged 19–26 years completed the task. All participants were right-handed and had no history of psychiatric or neurological disorders, and they were paid € 13 for their participation. Three participants were excluded for non-compliance with the instructions, and two because they already had previous knowledge of moral dilemmas. The final sample for behavioral and subjective data was thus composed of 51 participants (30 F, mean age = 22.40 years, *SD* = 1.71).

Participants were randomly assigned to a Trolley group (which was presented with primarily Trolley-type dilemmas) or a Footbridge group (which was presented with primarily Footbridge-type dilemmas), which were comparable for mean age and male/female ratio. Due to excessive EEG artifacts, eight additional participants were excluded, and the final sample for the EEG analysis was composed of 43 participants (Trolley group: *N* = 21, 11 F, mean age = 22.9 years, *SD* = 1.41; Footbridge group: *N* = 22, 12 F, mean age = 22.27 years, *SD* = 1.69).

### Stimuli

We used a set of 60 standardized dilemmas (Lotto et al., 2014) including 30 Footbridge-type dilemmas, which described killing one individual as an intended means to save others, and 30 Trolley-type dilemmas, which described killing one individual as a foreseen but unintended consequence of saving others^1^. All dilemmas were presented as written text on three consecutive slides: the *scenario* described the context, in which a threat endangers several people’s lives; *option A* described the non-utilitarian choice, in which the agent lets these people die; *option B* described the utilitarian choice, in which the agent kills one person to save these people. Additionally, one *counterfactual slide* was presented for each dilemma depending on the decision made by the participant. This slide described the consequences of the unchosen option in terms of number of deaths and lives (**Table 1**).

**Table 1:** Sample stimuli used in the task.

Sample Trolley- and Footbridge-type dilemmas.				

**Scenario**	**Option A (non-utilitarian)**	**Option B (utilitarian)**	**Counterfactual statement after choice “A”**	**Counterfactual statement after choice “B”**

**Trolley-type dilemma**				
You are a fireman who is trying to save some people trapped in a fire which has broken out on the 20th floor of a skyscraper, which is luckily deserted. Six people are trapped in a room on the 21st floor and are already beginning to suffocate from the smoke, while one person is coming down alone from the 19th floor on foot.	You let the fire reach the 21st floor of the skyscraper. The six people on that floor will die.	You activate the automatic closure of the fire doors. You know that this will mean that the person coming down will be unable to escape from the building. He will die, but it will give you time to save the other six.	If you had activated the closure, the person coming down from the building would be dead and the other six would be alive.	If you hadn’t activated the closure, the six people would be dead and the one that was coming down would be alive.

**Footbridge-type dilemma**				

You are carrying out research into the behavior of lions in the Savannah of Central Africa. From an observation tower, you can see four people hide behind a bush. They are being followed by a lion and are trying to get away from it. The lion has seen them and is heading for the bush. Another person has been able to climb the observation tower.	You let the ferocious lion reach the four people hidden behind the bush. They will be mauled to death.	You push off the person who has climbed the tower so that the lion is drawn toward him. You know that this person will be mauled to death, but the other four will have time to escape.	If you had pushed the person off the tower, the person on the tower would be dead and the four hiding behind the bush would be alive.	If you had not pushed the person off the tower, the four people behind the bush would be dead and the one on the tower would be alive.


*After reading the scenario and both options participant had to choose between options A and B. If they chose option A, the counterfactual statement for option B was presented. If they chose option B, the counterfactual statement for option A was presented.*

Four additional moral dilemmas, which involved no deaths and described other moral issues (e.g., stealing, lying, and being dishonest), were used as filler stimuli to avoid automaticity in the responses, and were not analyzed.

Stimulus presentation was accomplished with E-prime software (Psychology Software Tools Inc., 2012).

### Procedure

Upon arrival at the laboratory, participants read and signed an informed consent form and, after that the elastic cap for EEG recordings was applied, received instructions for the task. We chose not to administer the whole 60-dilemma set to each participant, because the task would have lasted more than 2 h and would have been excessively repetitive and tiring for the participants, thus compromising the reliability of their performance. However, to compute the Movement Related Potentials (MRPs), of which the RP is a component, a minimum of 30 trials per condition is needed. For this reason, participants in the Footbridge group were presented with 30 Footbridge-type dilemmas, 10 Trolley-type dilemmas, and four fillers; participants in the Trolley group were presented with 30 Trolley-type dilemmas, 10 Footbridge-type dilemmas, and four fillers. We then used Footbridge-type trials only to compute the RP for the Footbridge group, and Trolley-type trials only to compute the RP for the Trolley group (see Analysis for details).

**FIGURE 1:**
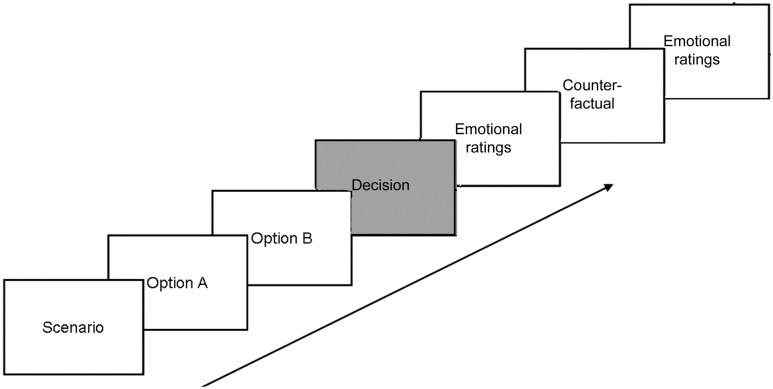
**Sequence of events in the experiment.** Participants had to decide between Options A and B by pressing the corresponding key during the presentation of the decision slide (in gray). Then, they had to rate how they felt after having chosen. Afterward, they were presented with a counterfactual slide describing what would have happened if they had chosen the alternative option. Finally, they had to rate how they would have felt if they had chosen the alternative option. Movement Related Potentials (MRPs) were recorded time-locked to the behavioral response, during the decision slide.

Dilemmas were divided in two blocks and presented in a pseudo-randomized order, so that each block comprised 15 dilemmas of the main category for the group, five dilemmas of the other category, and two fillers. In each trial, participants read the three text slides describing the scenario and the two options at their own pace and advanced by pressing the spacebar. Then, a fixation cross appeared on screen, and participants were instructed to decide between the two options by pressing one of two computer keys marked “A” and “B” with the index or the middle finger of the right hand. The fixation cross remained on the screen until participants responded, for a maximum time of 10 s, plus one additional second after the response (to prevent the MRPs to be contaminated by the event-related potentials associated with the offset of the slide). Then, participants rated how they felt after the decision on six 0–6 Likert scales indicating the intensity of six emotions: anger, disgust, guilt, *action* regret^2^, *inaction* regret, and shame. Participants were instructed to choose 0 when they did not experience the emotion at all, and 6 when they experienced the emotion at a maximal intensity. Subsequently, participants read the counterfactual slide, which described the consequences of the alternative, unchosen, option (**Table 1**) and rated how they would have felt if they had chosen the alternative option on the same six emotional scales, presented in random order (**Figure 1**). This procedure allowed us to collect for each trial an emotional state associated to the chosen option and an emotional state associated to the unchosen one. Moreover, by labeling the ratings according to the option to which they were associated, we also obtained for each trial a post-decisional emotional state, associated to the utilitarian option and one associated to the non-utilitarian option.

The stimuli were displayed on a 19 inches monitor at a viewing distance of 100 cm, and the experimental task started after three practice trials. The task lasted about 1 h, plus half-an-hour preparation time.

### Data Reduction and Analysis

Subjective and behavioral data were analyzed with mixed effect models, including all trials for each participants except the filler trials, those trials in which participants did not respond in time (total *N* = 33, maximum *N* per participant = 6), and one trial with a response time <120 ms, which was considered as an anticipation. For these analyses, as opposed to those performed on MRPs (see below), all dilemmas were included in the analysis for every participant, irrespective of the group. This was possible because behavioral and subjective data were analyzed using mixed effect models, which do not require the same number of observations for each cell of the design. For these analyses, the Dilemma Type factor was a within-participants variable, and the data were analyzed irrespective of participants’ group.

First of all, to investigate whether the emotions associated with the utilitarian and non-utilitarian options differed, and whether this difference was modulated by dilemma type, we built a separate mixed effect linear regression model for each emotion, with emotional intensity as dependent variable, the *Option* to which the emotion was associated (utilitarian, non-utilitarian), *Dilemma Type* (Trolley-type, Footbridge-type) and *Option* × *Dilemma Type* as fixed effects, and participant and item (i.e., the single dilemma) as random effects.

To investigate whether the type of dilemma influenced the probability of choosing the utilitarian option, we built a mixed effect logistic regression model with choice (0 = non-utilitarian, 1 = utilitarian) as dependent variable, *Dilemma Type* as fixed effect, and participant and item as random effects.

Finally, to investigate whether participants chose the option that was associated with the lowest emotional intensities and whether this effect was modulated by the type of dilemma, we calculated for each trial and each emotion a differential intensity index by subtracting the intensities associated with the non-utilitarian option from those associated with the utilitarian option. Then, we built mixed effect logistic regression models with choice as dependent variable, *Emotional Intensity Difference*, *Dilemma Type*, and *Emotional Intensity Difference* × *Dilemma Type* as fixed effects, and participant and item as random effects. Since we were interested in the specific effect of each single emotion, we calculated separate models for each emotion calculated trial by trial. For all these analyses, the emotional ratings were categorized based on the option to which they were associated, irrespective of whether they were provided after the choice or after the counterfactual statement. For each analysis, we started with the model including only the random effects and then introduced the fixed effects one by one, in the order described above. To compare models, we used the log-likelihood ratio test. To test the significance of parameters of the fixed effects, Wald *z* tests were used for logistic models and *t*-test with the Satterthwaite approximations for degrees of freedom for linear models.

As for electrophysiological data, the EEG was recorded from nine tin electrodes (Fz, Cz, Pz, F3, F4, C3, C4, P3, P4) embedded in an elastic cap and a tin electrode applied on the right mastoid (Electro-Cap International, Inc.; Eaton, OH, USA). All impedances were kept below 10 kΩ, and the left mastoid was used as reference. All sites were re-referenced off-line to the average of the left and right mastoids. Vertical and horizontal electro-oculogram were recorded from additional electrodes placed above and below the left eye and at the external canthi of both eyes, with the left mastoid as online reference and off-line bipolar re-referencing. The signal was amplified with a BrainVision V-Amp amplifier (Brain Products GmbH, Gilching, Germany), bandpass filtered (DC - 70 Hz) and digitized at 500 Hz (24 bit A/D converter, accuracy 0.04 uV per least significant bit). Blink artifacts and eye movements were corrected with a regression-based algorithm (Gratton et al., 1983). The EEG was epoched into 1500-ms segments, starting from 1000 ms before the keypress and ending 500 ms after. To correct for slow DC shifts, each epoch was linear detrended. Then, each epoch was re-filtered with a 30 Hz low pass filter (12 dB/oct) and baseline-corrected against the mean-voltage recorded during a 200-ms period from 1000 to 800 ms preceding keypress. Only epochs pertaining Trolley-type dilemmas were retained for the Trolley group, and vice versa for the Footbridge group. The epochs were then visually screened for artifact and each epoch containing a voltage higher than ±80 μV in any channel was rejected from further analysis. The remaining epochs were averaged separately for each participant (mean retained epochs for the Trolley group: 23.14, *SD*: 7.58; mean retained epochs for the Footbridge group: 22.98, *SD*: 7.68). The amplitude of the RP was measured in two time intervals (Shibasaki and Hallett, 2006): (1) mean negativity between 800 and 500 ms before keypress (*early* RP); and (2) mean negativity between 500 and 50 ms before keypress (*late* RP). Statistical analyses were restricted to Cz since the RP measured at this electrode reflects the activation of the SMA (Shibasaki and Hallett, 2006), and since in the study by Gluth et al. (2013) the potential recorded at Cz tracked the emergence of value-based decisions.

For all the analyses performed on RP amplitude, the Dilemma Type was a between-participants factor, since only Footbridge-type dilemmas were included in the analyses for the Footbridge group and only Trolley-type dilemmas were included in the analyses for the Trolley group.

To compare the amplitude of the RP between dilemma types, *T*-tests with Welch-corrected degrees of freedom were performed separately for each time window.

To test whether the amplitude of the RP reflected emotional conflict, an index of *emotional conflict* was calculated as the mean difference between emotional intensities associated to chosen vs. unchosen options, irrespective of whether they referred to the utilitarian or non-utilitarian option, averaged across the six emotions. Participants whose emotional conflict values were negative are supposed to have experienced lower emotional conflict during the task, since they tended to choose the option associated with the lowest intensities of negative emotions, thus following their emotion in their choice; participants whose conflict values were close to zero are supposed to have experienced greater conflict during the task, since they did not clearly prefer one option over another based on emotional intensities, and thus could not follow their emotion in their choice; finally, participants whose conflict values were positive are supposed to have experienced greater conflict since on average they chose the option associated with the most intense negative emotions, thus going directly against their emotion in their choice. Linear regressions were calculated separately for each emotion and time window, using the RP amplitude as dependent variable and the emotional conflict as predictor.

For both behavioral and electrophysiological data analyses we calculated the approximate Bayes Factor (BF) through the Bayesian Information Criterion (BIC), following the procedure described in Wagenmakers (2007), in order to provide further information on the probability of the effects given the data. This is especially useful in case of null results: in the framework of traditional null hypothesis testing, the failed rejection of the null hypothesis (H0) is uninformative because it does not allow to state whether the data actually support H0 or not. Conversely, the *BF* allow to assess the relative likelihood of the null and alternative (H1) hypotheses (Jarosz and Wiley, 2014). A *BF*_10_ greater than one implies that the data are more likely to occur under H1 than under H0. Similarly, a *BF*_10_ lower than one indicates that the data are more likely to occur under H0 than under H1. A *BF*_10_ = 3, for instance, means that the data are three times more likely to have occurred under H1 than under H0. Following the guidelines by Etz and Vandekerckhove (2016), *BF*_10_s between one and three are interpreted as ambiguous, between 3 and 10 as moderately in favor of H1, larger than 10 as strongly in favor of H1.

All statistical analysis were performed in R (R Core Team, 2015), using the libraries stats (R Core Team, 2015), lme4 (Bates et al., 2014), lmerTest (Kuznetsova et al., 2015) and effects (Fox, 1987).

## Results

### Emotional Intensity Ratings

**FIGURE 2:**
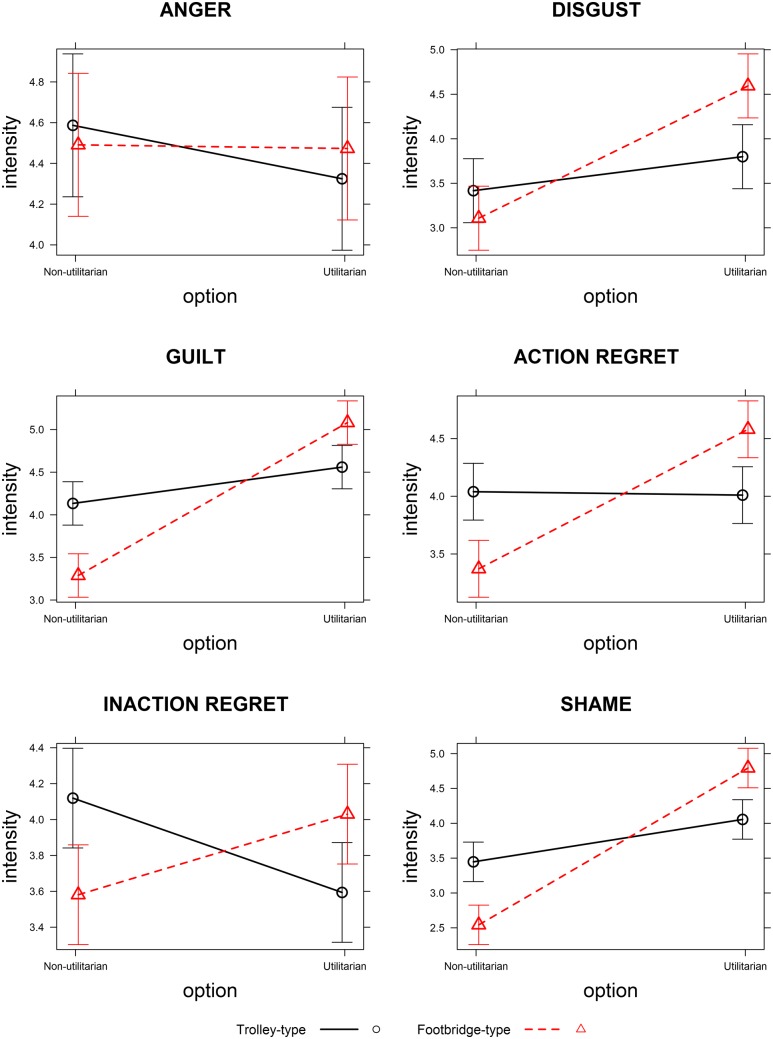
**Effects of Dilemma Type on emotional intensities as a function of Option.** The scales ranged from 0 (no intensity) to 6 (maximal intensity). Error bars indicate 95% confidence intervals.

For each emotion, the best model included the interaction between *Dilemma Type* and *Option*, which was always significant. The posterior probabilities showed positive to very strong evidence of the effect of option on emotional intensities being modulated by the type of dilemma. The statistical results are reported in **Table 2** and the effects are displayed in **Figure 2**.

**Table 2:** Linear regression mixed effects models depicting the Option × Dilemma Type interactions on emotional intensities.

Results on emotional intensity ratings				

**Emotion**	**Model**	**Log-likelihood ratio test**	**Bayes Factor and posterior probabilities**	**Fixed effects parameters**
Anger	Option × Dilemma type	χ^2^(1) = 11.42, *p* < 0.001	*BF*_10_ ≈ 4,76 Pr(*H_1_|D*) ≈ 83%	Option: *B* = -0.26, *SE* = 0.05, *t*(3924) = -5.14, *p* < 0.001 Dilemma Type: *B* = -0.10, *SE* = 0.06, *t*(127) = -1.48, *p* = 0.14 Option × Dilemma Type: *B* = 0.24, *SE* = 0.07, *t*(3924) = 3.38, *p* < 0.001
Disgust	Option × Dilemma type	χ^2^(1) = 147.45, *p* < 0.001	*BF*_10_ > 150 Pr(*H_1_|D*) ≈ 99%	Option: *B* = 0.38, *SE* = 0.06, *t*(3923) = 6.00, *p* < 0.001 Dilemma type: *B* = -0.31, *SE* = 0.08, *t*(122) = -3.62, *p* < 0.001 Option × Dilemma Type: *B* = 1.10, *SE* = 0.09, *t*(3923) = 12.25, *p* < 0.001
Guilt	Option × Dilemma type	χ^2^(1) = 222.49, *p* < 0.001	*BF*_10_ > 150 Pr(*H_1_|D*) ≈ 99%	Option: *B* = 0.43, *SE* = 0.06, *t*(3928) = 6.67, *p* < 0.001 Dilemma Type: *B* = -0.84, *SE* = 0.10, *t*(116) = -8.57, *p* < 0.001 Option × Dilemma Type: *B* = 1.36, *SE* = 0.09, *t*(3928) = 15.13, *p* < 0.001
Action regret	Option × Dilemma type	χ^2^(1) = 199.21, *p* < 0.001	*BF*_10_ > 150 Pr(*H_1_|D*) ≈ 99%	Option: *B* = -0.03, *SE* = 0.06, *t*(3923) = -0.48, *p* = 0.63 Dilemma Type: *B* = -0.67, *SE* = 0.08, *t*(121) = -8.19, *p* < 0.001 Option × Dilemma Type: *B* = 1.24, *SE* = 0.09, *t*(3923) = 14.29, *p* < 0.001
Inaction regret	Option × Dilemma type	χ^2^(1) = 118.44, *p* < 0.001	*BF*_10_ > 150 Pr(*H_1_|D*) ≈ 99%	Option: *B* = -0.53, *SE* = 0.06, *t*(3922) = -8.38, *p* < 0.001 Dilemma Type: *B* = -0.54, *SE* = 0.07, *t*(124) = -6.97, *p* < 0.001 Option × Dilemma Type: *B* = 0.97, *SE* = 0.09, *t*(3922) = 10.96, *p* < 0.001
Shame	Option × Dilemma type	χ^2^(1) = 262.6, *p* < 0.001	*BF*_10_ > 150 Pr(*H_1_|D*) ≈ 99%	Option: *B* = 0.61, *SE* = 0.07, *t*(3925) = 8.65, *p* < 0.001 Dilemma Type: *B* = -0.9, *SE* = 0.1, *t*(121) = -9.14, *p* < 0.001 Option × Dilemma Type: *B* = 1.62, *SE* = 0.1, *t*(3925) = 16.47, *p* < 0.001


*The log-likelihood ratio test and the Bayes Factors (BFs) compare the depicted models with the models including Option and Dilemma Type, but no interaction. The factor Option was dummy-coded so that non-utilitarian options were 0 and utilitarian options were 1. The factor Dilemma Type was dummy-coded so that Trolley-type dilemmas were 0 and Footbridge-type dilemmas were 1.*

Follow-up analysis performed separately on Trolley-type and Footbridge-type dilemmas (**Tables 3** and **4**, respectively) showed that, as concerns anger, the interaction effect was due to lower emotional intensities reported for utilitarian as compared to non-utilitarian options in Trolley-type dilemmas only. As concerns disgust, guilt and shame, the interaction was due to higher emotional intensities reported for utilitarian as compared to non-utilitarian options for both dilemma types, and to this difference being more pronounced for Footbridge-type as compared to Trolley-type dilemmas. As concerns action regret, the interaction was due to higher emotional intensities for utilitarian as compared to non-utilitarian options in Footbridge-type dilemmas only. For inaction regret, the interaction was due to lower emotional intensities for utilitarian as compared to non-utilitarian options in Trolley-type dilemmas and to higher emotional intensities for utilitarian as compared to non-utilitarian options in Footbridge-type dilemmas.

**Table 3:** Linear regression mixed effects models depicting the effects of Option on emotional intensities in Trolley-type dilemmas.

Results on emotional intensity ratings – Trolley-type dilemmas only				

**Emotion**	**Model**	**Log-likelihood ratio test**	**Bayes Factor and posterior probabilities**	**Fixed effects parameters**
Anger	Option	χ^2^(1) = 25.44, *p* < 0.001	*BF*_10_ > 150 Pr(*H_1_|D*) ≈ 99%	Option: *B* = -0.26, *SE* = 0.05, *t*(1944) = -5.06, *p* < 0.001
Disgust	Option	χ^2^(1) = 36.58, *p* < 0.001	*BF*_10_ > 150 Pr(*H_1_|D*) ≈ 99%	Option: *B* = 0.38, *SE* = 0.06, *t*(1947) = 6.07, *p* < 0.001
Guilt	Option	χ^2^(1) = 48.55, *p* < 0.001	*BF*_10_ > 150 Pr(*H_1_|D*) ≈ 99%	Option: *B* = 0.43, *SE* = 0.06, *t*(1948) = 7.01, *p* < 0.001
Action regret	Option	χ^2^(1) = 0.25, *p* = 0.62	*BF*_10_ ≈ 0.02 Pr(*H_1_|D*) ≈ 2%	Option: *B* = -0.03, *SE* = 0.06, *t*(1945) = -0.5, *p* = 0.62
Inaction regret	Option	χ^2^(1) = 76.98, *p* < 0.001	*BF*_10_ > 150 Pr(*H_1_|D*) ≈ 99%	Option: *B* = -0.53, *SE* = 0.06, *t*(1945) = -8.86, *p* < 0.001
Shame	Option	χ^2^(1) = 79.09, *p* < 0.001	*BF*_10_ > 150 Pr(*H_1_|D*) ≈ 99%	Option: *B* = 0.61, *SE* = 0.07, *t*(1949) = 8.98, *p* < 0.001


*The log-likelihood ratio test and the BFs compare the depicted models with the models including only the random effects. The factor Option was dummy-coded so that non-utilitarian options were 0 and utilitarian options were 1.*

**Table 4:** Linear regression mixed effects models depicting the effects of Option on emotional intensities in Footbridge-type dilemmas.

Results on emotional intensity ratings – Footbridge-type dilemmas only				

**Emotion**	**Model**	**Log-likelihood ratio test**	**Bayes Factor and posterior probabilities**	**Fixed effects parameters**
Anger	Option	χ^2^(1) = 0.13, *p* = 0.72	*BF*_10_ ≈ 0.02 Pr(*H_1_|D*) ≈ 2%	Option: *B* = -0.02, *SE* = 0.05, *t*(1929) = -0.36, *p* = 0.72
Disgust	Option	χ^2^(1) = 482.17, *p* < 0.001	*BF*_10_ > 150 Pr(*H_1_|D*) ≈ 99%	Option: *B* = 1.48, *SE* = 0.06, *t*(1928) = 23.40, *p* < 0.001
Guilt	Option	χ^2^(1) = 620.97, *p* < 0.001	*BF*_10_ > 150 Pr(*H_1_|D*) ≈ 99%	Option: *B* = 1.79, *SE* = 0.07, *t*(1930) = 27.06, *p* < 0.001
Action regret	Option	χ^2^(1) = 342.72, *p* < 0.001	*BF*_10_ > 150 Pr(*H_1_|D*) ≈ 99%	Option: *B* = 1.21, *SE* = 0.06, *t*(1925) = 19.36, *p* < 0.001
Inaction regret	Option	χ^2^(1) = 46.14, *p* < 0.001	*BF*_10_ > 150 Pr(*H_1_|D*) ≈ 99%	Option: *B* = 0.49, *SE* = 0.06, *t*(1924) = 6.83, *p* < 0.001
Shame	Option	χ^2^(1) = 801.59, *p* < 0.001	*BF*_10_ > 150 Pr(*H_1_|D*) ≈ 99%	Option: *B* = 2.25, *SE* = 0.07, *t*(1929) = 31.52, *p* < 0.001


*The log-likelihood ratio test and the BFs compare the depicted models with the models including only the random effects. The factor Option was dummy-coded so that non-utilitarian options were 0 and utilitarian options were 1.*

### Choices

The Dilemma Type effect on choices was significant (*B* = -2.77, *SE* = 0.26, *z* = -10.51, *p* < 0.001; χ^2^(1) = 65.38, *p* < 0.001): the probability of choosing the utilitarian option was higher in Trolley-type as compared with Footbridge-type dilemmas (0.82 in Trolley-type dilemmas, 95% *CI* = [0.75, 0.88]; 0.23 in Footbridge-type dilemmas, 95% *CI* = [0.16, 0.32]). As indicated by the approximate Bayes Factor (*BF*_10_ > 150) the posterior probability of choices being modulated by dilemma type was Pr(*H_1_*|*D*) ≈ 99%.

The model that included as predictors the emotional intensity difference indexes calculated for each of the six emotions was significant [χ^2^(6) = 200.89, *p* < 0.001, *BF*_10_ > 150, Pr(*H_1_*|*D*) ≈ 99%), indicating that emotions did play a role in driving choices. Crucially, including Dilemma Type in the model significantly improved it [χ^2^(1) = 49.09, *p* < 0.001, *BF*_10_ > 150, Pr(*H_1_*|*D*) ≈ 99%; Dilemma type effect: *B* = -2.34, *SE* = 0.27, *z* = -8.51, *p* < 0.001], showing that differences in emotional intensities do not fully explain the difference in choices for the two dilemma types.

To further investigate the effect of each emotion on choices, we calculated an additional set of model separately for each emotion.

For each emotion, including emotional intensity difference in the model with only the random effects significantly improved it [guilt: χ^2^(1) = 84.31, *p* < 0.001; disgust: χ^2^(1) = 57.07, *p* < 0.001; anger: χ^2^(1) = 64.01, *p* < 0.001; action regret: χ^2^(1) = 56.61, *p* < 0.001; inaction regret: χ^2^(1) = 61.49, *p* < 0.001; shame: χ^2^(1) = 52.67, *p* < 0.001], and the posterior probability of choices being influenced by differential intensity were Pr(*H_1_*|*D*) ≈ 99% (*BFs*_10_ > 150) for every emotion. Introducing Dilemma Type significantly improved the models, and the approximate BFs indicated strong evidence for choices being influenced by the type of dilemma in addition to emotional intensity difference (**Table 5**). The models with the interaction effect did not significantly differ from the models with the two main effects [guilt: χ^2^(1) = 3.79, *p* = 0.05; disgust: χ^2^(1) = 1.92, *p* = 0.16; anger: χ^2^(1) = 0.001, *p* = 0.97; action regret: χ^2^(1) = 0.36, *p* = 0.55; inaction regret: χ^2^(1) = 0.17, *p* = 0.68; shame: χ^2^(1) = 0.42, *p* = 0.52]. For guilt, the interaction term was significant (*B* = -0.13, *SD* = 0.07, *z* = -2.0, *p* = 0.049), but the posterior probability of dilemma type modulating the effect of differential guilt intensities on choices was only Pr(*H_1_*|*D*) ≈ 13% (*BF*_10_ ≈ 0.15), and thus the significant interaction was probably a spurious effect. For the other emotions, the interaction term was non-significant (all *p*s > 0.15) and the posterior probability of the interaction was Pr(*H_1_*|*D*) ≈ 5% (*BF*_10_ ≈ 0.06) for disgust, Pr(*H_1_*|*D*) ≈ 2%, *BF*_10_ ≈ 0.02 for anger, Pr(*H_1_*|*D*) ≈ 3%, *BF*_10_ ≈ 0.03 for action regret, Pr(*H_1_*|*D*) ≈ 6%, *BF*_10_ ≈ 0.06 for inaction regret, and Pr(*H_1_*|*D*) ≈ 2%, *BF*_10_ ≈ 0.02 for shame.

**Table 5:** Logistic regression models depicting the effects of Emotional Intensity Difference (EID) and Dilemma Type on the probability of choosing the utilitarian option.

Effects of EID and Dilemma Type on utilitarian choices				

**Emotion**	**Model**	**Log-likelihood ratio test**	**Bayes Factor and posterior probabilities**	**Fixed effects parameters**
Anger	EID + Dilemma Type	χ^2^(1) = 64.01, *p* < 0.001	*BF*_10_ > 150 Pr(*H_1_|D*) ≈ 99%	EID: *B* = -0.18, *SE* = 0.04, *z* = -4.12, *p* < 0.001 Dilemma Type: *B* = -2.74, *SE* = 0.24, *z* = -10.35, *p* < 0.001
Disgust	EID + Dilemma Type	χ^2^(1) = 57.07, *p* < 0.001	*BF*_10_ > 150 Pr(*H_1_|D*) ≈ 99%	EID: *B* = -0.27, *SE* = 0.04, *z* = -7.41, *p* < 0.001 Dilemma Type: *B* = -2.58, *SE* = 0.27, *z* = -9.48, *p* < 0.001
Guilt	EID + Dilemma Type	χ^2^(1) = 53.51, *p* < 0.001	*BF*_10_ > 150 Pr(*H_1_|D*) ≈ 99%	EID: *B* = -0.30, *SE* = 0.04, *z* = -8.30, *p* < 0.001 Dilemma Type: *B* = -2.51, *SE* = 0.28, *z* = -9.06, *p* < 0.001
Action regret	EID + Dilemma Type	χ^2^(1) = 56.61, *p* < 0.001	*BF*_10_ > 150 Pr(*H_1_|D*) ≈ 99%	EID: *B* = -0.37, *SE* = 0.04, *z* = -9.83, *p* < 0.001 Dilemma Type: *B* = -2.51, *SE* = 0.27, *z* = -9.41, *p* < 0.001
Inaction regret	EID + Dilemma Type	χ^2^(1) = 61.49, *p* < 0.001	*BF*_10_ > 150 Pr(*H_1_|D*) ≈ 99%	EID: *B* = -0.38, *SE* = 0.04, *z* = -10.58, *p* < 0.001 Dilemma Type: *B* = -2.59, *SE* = 0.26, *z* = -9.99, *p* < 0.001
Shame	EID + Dilemma Type	χ^2^(1) = 52.67, *p* < 0.001	*BF*_10_ > 150 Pr(*H_1_|D*) ≈ 99%	EID: *B* = -0.28, *SE* = 0.03, *z* = -8.47, *p* < 0.001 Dilemma Type: *B* = -2.47, *SE* = 0.27, *z* = -8.94, *p* < 0.001


*The log-likelihood ratio test and the BFs compare the depicted models with the models including only EID as fixed effect.*

**FIGURE 3:**
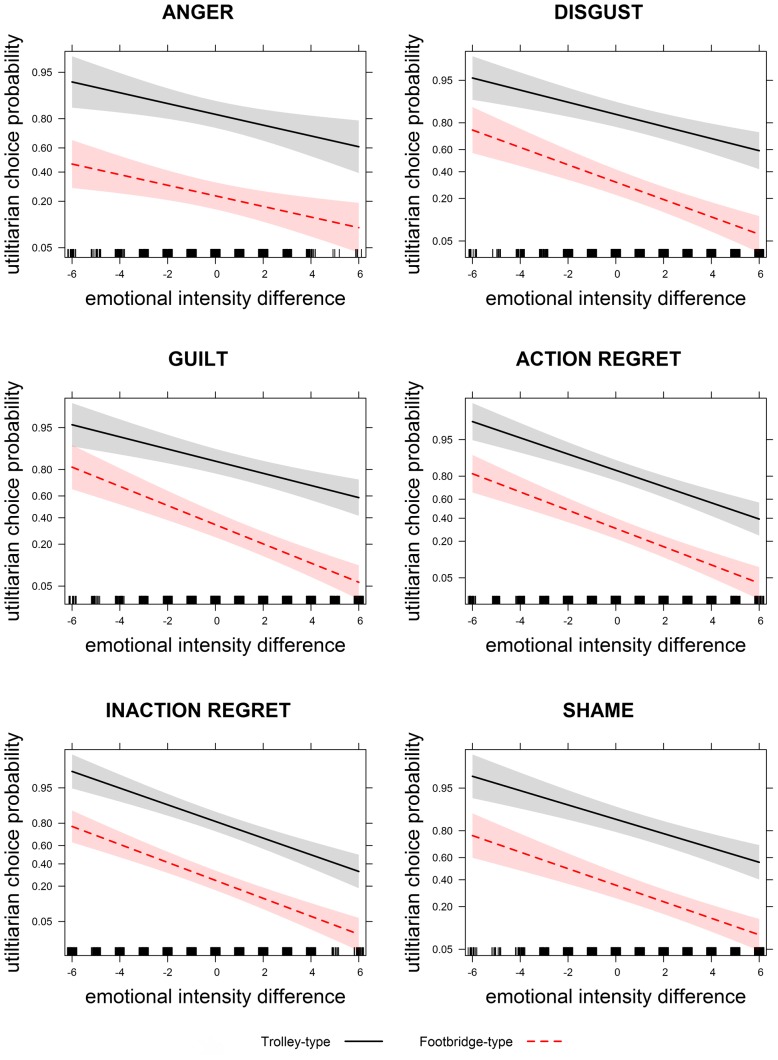
**Relationship between emotional intensity difference and probability of choosing the utilitarian option, represented separately for dilemma type.** Positive emotional intensity differences indicate that the utilitarian option was associated with stronger intensities than the non-utilitarian option. Negative emotional intensity differences indicate that the non-utilitarian option was associated with stronger emotional intensities than the utilitarian option. Shaded areas indicate 95% confidence intervals. The unequal spacing of the ticks in the *y* axes are because graphs are plotted on the logit scale, but the y axes are labeled on the scale of the probability of choosing the utilitarian option, after conversion from the logit scale.

Thus, for each emotion the difference in emotional intensities between the utilitarian and the non-utilitarian options was negatively associated with the probability to choose the utilitarian option: the greater the emotional intensities for the utilitarian option as compared to the non-utilitarian option, the lower the probability to choose the utilitarian option, and vice versa. This effect was not influenced by the type of dilemma (**Figure 3**).

### Electrophysiological Data

**FIGURE 4:**
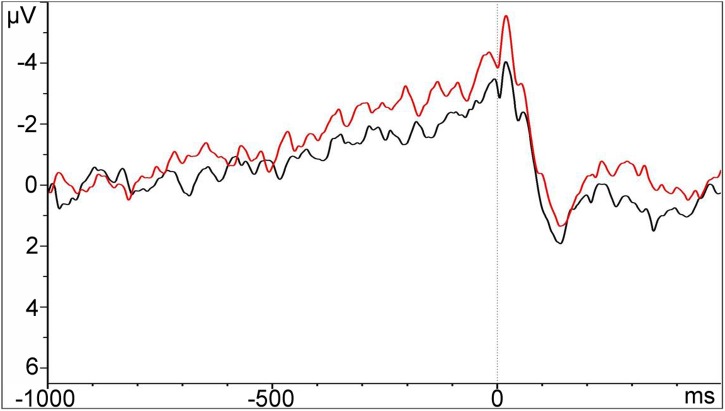
**Grand-averaged MRPs recorded at Cz time-locked to the behavioral response (choice) in the Trolley and Footbridge groups.** Time 0 indicates the onset of the behavioral response.

Grand-averaged MRPs recorded at Cz before choice in the Footbridge and Trolley groups are displayed in **Figure 4**.

### Early Readiness Potential

The Dilemma Type effect was not significant: the amplitude of the early RP did not differ between the Footbridge and the Trolley groups [*t*(40.71) = 1.17, *p* = 0.25]. The approximate BF indicates ambiguous evidence in favor of a difference between groups (*BF*_10_ ≈2.02, Pr(*H_1_*|*D*) ≈ 0.67).

As concerns the influence of emotional conflict on the amplitude of the early RP, the model was not significant (*R*^2^ = 0.01, *p* = 0.51, β = -0.14, *p* = 0.51). The approximate BFs indicate no clear evidence in favor of either the alternative or the null hypothesis [*BF*_10_ ≈1.26, Pr(*H_1_*|*D*) ≈ 0.56].

### Late Readiness Potential

Similar results were obtained for the late RP. The comparison between the Footbridge and Trolley groups was not significant [*t*(39.35) = 1.3, *p* = 0.20], and there was weak evidence in favor of a difference between groups [*BF*_10_ ≈ 2.36, Pr(*H1*|*D*) ≈ 0.70]. As for the previous time-window, the model investigating the effect of emotional conflict on the amplitude of the late RP was not significant (*R*^2^ = 0.03, *p* = 0.30, β = -0.36, *p* = 0.30). The approximate BFs indicate ambiguous evidence in favor of an effect of emotional conflict on RP amplitude (*BF*_10_ ≈ 1.75, Pr(*H1*|*D*) ≈ 0.64).

## Discussion

The main aim of this study was to investigate the role of anticipated post-decisional emotions in driving decisions in moral dilemmas. Through self-report ratings, we measured the emotional state experienced by participants both after their choice and after imagining to have chosen the alternative option. The emotional state was measured on six emotions that we hypothesized to be relevant in the resolution of this kind of dilemmas: *regret* [specified as action and inaction regret, according to the different terms used in Italian (Giorgetta et al., 2012)], *guilt*, *shame*, *anger*, and *disgust*. Thus, for every dilemma we collected the intensity of these emotions twice, one for each option (utilitarian and non-utilitarian), and we analyzed it irrespective of what participants decided. We hypothesized that, if individuals spontaneously anticipate the emotions they would feel after the decision and use this information as input in the decision process, then the difference between the emotional states related to the two alternatives, i.e., the utilitarian and the non-utilitarian option, would predict participant’s choices. As a second aim of the study, we investigated whether the RP might reflect emotional conflict in the context of moral dilemmas.

As for the behavioral data, we found two main results. First, utilitarian and non-utilitarian options elicited different emotional intensities as a function of the two dilemma types, with most emotions highlighting larger differences for Footbridge- than Trolley-type dilemmas. Second, the difference between emotional intensities associated with the two options predicted choices irrespective of dilemma type, suggesting that participants chose the option with the least emotional cost even in Trolley-type dilemmas.

As for the EEG data, our results were inconclusive, as they did not provide strong evidence either in favor or against our hypothesis. The results will be discussed in detail in the following paragraphs.

According to Greene et al. (2001, 2004) dual process model utilitarian options are rejected in Footbridge-type dilemmas because they evoke strong aversive emotional reactions. Thus, we hypothesized that, in Footbridge-type dilemmas, utilitarian options would be associated with higher negative emotional consequences as compared to non-utilitarian options. In Trolley-type dilemmas, in contrast, we anticipated the difference between the emotional consequences of the two options to be smaller, since according to Greene et al.’s (2001, 2004) model the utilitarian option should not elicit such strong emotional reactions in these dilemmas.

Results on emotional intensities of *guilt*, *shame*, and *disgust* were largely consistent with our hypothesis: the intensity of these emotions was higher for the utilitarian choices as compared to the non-utilitarian choices in Footbridge-type dilemmas, and this difference was significantly lower (but still present) for Trolley-type dilemmas. Thus, sacrificing one person to save more people (utilitarian option) elicits more intense self-condemning emotions than letting some people die (non-utilitarian option), and this effect is especially pronounced when the sacrifice is performed intentionally (i.e., as a means to an end), as in Footbridge-type dilemmas. Sacrificing one person intentionally also elicited more action regret than letting some people die, whereas sacrificing one person as a side effect, as in Trolley-type dilemmas, did not, in line with an account of regret as being strongly influenced by agency and personal responsibility (e.g., Zeelenberg et al., 2000; Giorgetta et al., 2012; Wagner et al., 2012).

Results on *anger* and inaction regret, on the other hand, followed a different trend. *Anger* was stronger for non-utilitarian options in Trolley-type dilemmas only (albeit this effect was weak), with no difference emerging for Footbridge-type dilemmas. Thus, the intensity of anger was reduced by choosing the utilitarian option, as compared to choosing the non-utilitarian option. This is in line with results reported by Choe and Min (2011), who showed that high trait anger was positively associated with utilitarianism, and by Ugazio et al. (2012), who showed that inducing anger in participants before a moral dilemma task, increased the percentage of utilitarian choices. This positive relationship between anger and the utilitarian choice could be due to the fact that anger is an approach-related emotion entailing a motivation to act, and the utilitarian choice in moral dilemmas entails action, whereas the non-utilitarian choice entails inaction. Consistent with this interpretation, we found *inaction regret* to be stronger for the non-utilitarian option as compared to the utilitarian one in Trolley-type dilemmas. These results importantly indicate that in the case of Trolley-type dilemmas, choosing the utilitarian option is not only backed up by a rational cost-benefit analysis (e.g., Greene et al., 2001, 2004), but also by the need to avoid stronger feelings of inaction regret and anger. This is in line with other results reporting a positive relationship between emotional activation and utilitarian choices in Trolley-type dilemmas (Patil et al., 2014).

The fact that in Footbridge-type dilemmas inaction regret was higher for the utilitarian than for the non-utilitarian option might seem counterintuitive. Whereas in Trolley-type dilemmas inaction regret seems to be especially elicited by the negative consequences caused by inaction (letting some people die, in Footbridge-type dilemmas this emotion closely follows the pattern observed for guilt, shame, disgust, and action regret), and seems to be more strongly elicited by the aversive impact of intentional killing, which entails action rather than inaction. Further research should address what factors might modulate the intensity of this emotion.

Crucially, as concerns the effect of emotion on choices, our data are in line with the hypothesis that individuals choose the option with the lower anticipated emotional consequences: for each emotion, the difference in emotional intensity between utilitarian and non-utilitarian options was significantly associated with choices, indicating that participants chose the option with the least aversive emotional consequences. Interestingly, however, this effect was not modulated by the type of dilemma: not only in Footbridge-type dilemmas, as can be hypothesized based on the dual process theory (Greene et al., 2004, 2001), but also in Trolley-type dilemmas choices were influenced by emotion. This differs from what emerged in Tasso et al. (unpublished), who reported a significant association between emotional intensities and choices for Footbridge-type dilemmas only. However, Tasso et al. (unpublished) only analyzed those trials in which participants provided typical choices (i.e., utilitarian in Trolley-type dilemmas and non-utilitarian in Footbridge-type dilemmas). In contrast, the present study included all trials in the analyses, thus providing a more complete picture of the mechanisms at play.

Our results indicate that the option associated to the least intense negative emotions had a higher probability of being chosen, and this effect had the same magnitude in both dilemma types. However, based on the analyses on emotional ratings, we found that in Footbridge-type dilemmas the utilitarian option elicited overall higher emotional intensities as compared to the non-utilitarian one. This effect was observed for all the emotions that we tested, except for anger, which had comparable intensities for the two options. On the other hand, in Trolley-type dilemmas, the intensity of anger and inaction regret was higher for the non-utilitarian than the utilitarian option, whereas the intensity of disgust, guilt, and shame was higher for the utilitarian than for the non-utilitarian one, with these differences being smaller than those emerged for Footbridge-type dilemmas. Thus, our data suggest that, even though anticipating post-decisional emotions influenced choice probability in both Trolley- and Footbridge-type dilemmas, it is more likely that individuals choose the non-utilitarian option in Footbridge- than in Trolley-type dilemmas, because it elicited on average stronger emotional intensities in the former dilemmas than in the latter.

Our data on the effect of emotion on choice provided another important result: with emotional intensities held constant, the probability of choosing the utilitarian option was higher in Trolley-type than in Footbridge-type dilemmas. Thus, the different probability of choosing the utilitarian option that emerges between Trolley-type and Footbridge-type dilemmas cannot be exclusively attributed to differences in emotional intensities between dilemma types. This is in line with what reported by Horne and Powell (2016), who found that emotional reactions only partially explained the difference in moral judgment between Trolley-type and Footbridge-type dilemmas.

Several additional process can be hypothesized to account for the residual difference. First, drawing again from the dual process model (Greene et al., 2001, 2004), we can hypothesize that Trolley-type dilemmas, as compared to Footbridge-type dilemmas, activate more strongly cognitive processes such as reflection and reasoning, which would favor the utilitarian resolution.

Another relevant factor that could differentiate between dilemma types might be the representation of rules. According to the studies of Nichols (2002) and Nichols and Mallon (2006), individuals’ moral judgments are based not only on the emotional reactions elicited by the outcomes of an action, but also on a *normative theory* – that is, a body of norms describing what is allowed and what is not that would be acquired during the development (Nichols, 2002). As is the case for other types of rules, it can be hypothesized that moral rules are stored in long-term memory (Bunge, 2004). Even though emotional reactions play an important role in the formation and implementation of moral norms (e.g., Haidt, 2001; Greene, 2008; Buckholtz and Marois, 2012), it might be the representation of a rule against killing that contribute to the difference in responding to Footbridge- and Trolley-type dilemmas. Since killing a person in Footbridge-type dilemmas is perceived as more intentional than in Trolley-type dilemmas, the rule violation would be more severe in Footbridge-type dilemmas (cf. Cushman and Young, 2011). This might reduce the probability of choosing the utilitarian option.

As a third possibility, the difference in the probability of choosing the utilitarian option in the two dilemma types might be due to other emotions that we did not measure in the present study (e.g., empathy for the victims). Previous literature showed that the disposition to feel empathic concern and personal distress in front of the suffering of others was inversely associated with the endorsement of the utilitarian option (Gleichgerrcht and Young, 2013; Sarlo et al., 2014; Spino and Cummins, 2014; Patil et al., 2016). Personal distress, in particular, predicted the percentage of utilitarian choices in Footbridge-type, but not in Trolley-type dilemmas (Sarlo et al., 2014). Moreover, the present study did not investigate whether the emotions reported by participants were elicited by the outcome of the action (i.e., the victims) or by the harmful action itself (i.e., killing). A recent theoretical model of moral decision posits that these two sources of emotions are differentially engaged by different dilemma types, with Trolley-type dilemmas eliciting more outcome-based emotions and Footbridge-type dilemmas more action-based emotions (Cushman et al., 2012; Miller et al., 2014). It would be interesting in future studies to ask participants to specifically report emotions elicited by these different features of decision-making, in order to disentangle their effects on choices and to determine how they are modulated by dilemma types.

Finally, it is important to stress that in our study the self-report measures only captured the hypothetical emotional consequences that participants were aware of, leaving out the unconscious emotional reactions developing during or after decision-making. This is a crucial point, because affective reactions do not need to reach awareness in order to influence decisions and behaviors (e.g., Damasio, 1994). Thus, this research only captured a part of the emotional states elicited by the resolution of moral dilemmas – that is, those that participants consciously perceived – and might have underestimated the effect of emotion on decisions, and their impact in differentiating between Trolley-type and Footbridge-type dilemmas

As for the electrophysiological data, in the present study the amplitude of the RP, as opposed to previous results reported by Sarlo et al. (2012), did not discriminate between Trolley-type and Footbridge-type dilemmas, with the BF indicating weak evidence in favor of a difference. This might be due to the fact that, as opposed to Sarlo et al. (2012), we analyzed the RP using a between-participants design, which increased the variability of the data, thus reducing statistical power.

Also, we did not find concrete evidence pointing toward a relationship between the RP amplitude and the emotional conflict index, which was calculated by averaging the differences between the emotional intensity associated with the unchosen vs. the chosen one. The lack of any relationship might indicate that anticipated emotions did not influence the neural correlates of the last phase of decision-making, but possibly played a role in the earlier stages. As an alternative, it might indicate that the RP, in this context, does not reflect an emotional conflict, but a more general form of conflict arising from different processes. Future research might devise a more comprehensive measure of conflict – measuring, for instance, cognitive preferences in addition to emotional consequences – and test its relationship with the RP amplitude. In any case, it is also important to point out that the BFs for these null results were close to one, indicating almost equal probability between the null hypothesis and the alternative one. For this reason, we cannot exclude the possibility that a relationship between the RP amplitude and the emotional conflict does exist, but did not emerge in the present data due to lack of statistical power. Thus, the functional role of the RP in the context of moral decisions and its relationship to conflict and to emotional processing requires further investigation.

As a main limitation of the present study, it is important to stress that in our paradigm we did not measure anticipated emotions directly. Rather, we measured post-decisional and counterfactual emotions, and hypothesized participants to spontaneously anticipate these emotions during the decision. Some studies indicate that individuals are not always accurate in predicting how they would feel after making a choice, and that there is often a discrepancy between anticipated emotions and actual post-decisional emotions (Wilson and Gilbert, 2005). In the context of moral dilemmas, however, all the decisions that participants made are hypothetical, and participants are not confronted with real consequences. For this reason, we can expect post-decisional emotional ratings to reflect the emotional consequences that participants anticipated while they were making their choices. However, it cannot be excluded that the decision itself influenced post-decisional emotional evaluations. In any case, if we asked participants to report anticipated emotions before the decision, we would have probably biased participants to take emotions into account more than they would have done spontaneously, and thus our findings on choices would have been altered. Thus, we believe our paradigm was a good compromise allowing the study of the role played by anticipated emotions in moral dilemmas without generating considerable modifications to the decision process itself.

The results reported in this study indicate that in moral dilemmas participants choose the option that minimized the intensity of the aversive emotions experienced after the decision. This effect was present in both dilemma types and did not eliminate the differences in the probability of choosing the utilitarian option for the two dilemma types. This study thus provides useful indications for the understanding of how emotions influence the resolution of moral dilemmas. Future studies should investigate if the difference between Trolley- and Footbridge-type dilemmas that is not explained by anticipated emotions is due to the contribution of reflection and reasoning, rules, unconscious emotional reactions, or to an interplay between these factors.

## Ethics Statement

This study was approved by Comitato Etico della Ricerca Psicologica. Participants were informed of the duration of the task, they were informed about the fact that it was a computerized task in which the electroencephalogram was recorded, they were informed about the procedure involved for the acquisition of the encephalogram. They were informed that they could quit the task and leave at any time they saw fit without any penalty, and obtaining the full destruction of all their data. They were informed that their data would remain confidential and coded so that no detail that could allow their identification would appear in the dataset.

## Author Contributions

LL, AT, MS, and CP designed the study; CP acquired the data and performed the analysis; CP, LL, AT, and MS interpreted the results; CP wrote the paper; LL, AT, and MS critically revised the paper, all authors approved the final version for publication and agreed to be accountable for all aspects of the work in ensuring that questions related to the accuracy or integrity of any part of the work are appropriately investigated and resolved.

## Conflict of Interest Statement

The authors declare that the research was conducted in the absence of any commercial or financial relationships that could be construed as a potential conflict of interest.

## Footnotes

1 This dilemma set differs from the “personal-impersonal” dilemma set originally developed by Greene et al. (2001, 2004) in a few key respects. Here, the main criterion according to which the dilemmas are divided into Footbridge- or Trolley-type categories is based on the intentional structure of the proposed actions. Furthermore, several confounding factors have been excluded, as these dilemmas only address the issues of killing and letting die, rather than other moral issues, and do not include children, friends, or relatives in the scenarios. Lastly, mean number of words and number of text characters have been fully balanced between the two categories. Detailed information on the criteria used to develop this set of stimuli can be found in Sarlo et al. (2012) and in Lotto et al. (2014).

2 We employed both “action” and “inaction” regret because the emotion of regret does not have an univocal translation in the Italian language: in Italian, regret can be translated into three main words: *rimorso*, which is more related to action; *rimpianto*, which is more related to inaction, and *rammarico*, which is generally related to bad outcomes (Giorgetta et al., 2012). We chose to focus on the first two emotional labels, because they are differentially influenced by agency and responsibility (Giorgetta et al., 2012), and thus can be hypothesized to be differentially elicited by the utilitarian and non-utilitarian options in moral dilemmas.
